# Granulomas in the Heart: Pathophysiology, Diagnosis, Management, and Outcomes in Cardiac Sarcoidosis

**DOI:** 10.7759/cureus.100565

**Published:** 2026-01-01

**Authors:** Mayar B Alnasralla, Balaji Subbaraman

**Affiliations:** 1 Cardiology, Royal Private English School, Fujairah, ARE; 2 Interventional Cardiology, Al Sharq Hospital, Fujairah, ARE

**Keywords:** cardiac magnetic resonance (cmr), cardiac sarcoidosis (cs), conduction system disease, granulomatous myocarditis, heart failure, immunosuppressive therapy, myocardial fibrosis, sarcoidosis, ventricular arrhythmias (vas)

## Abstract

Cardiac sarcoidosis (CS) is a rare but potentially fatal manifestation of systemic sarcoidosis, characterized by granulomatous myocardial inflammation that disrupts both cardiac structure and electrical conduction. This narrative review synthesizes current understanding of CS pathophysiology, including immune dysregulation, CD4⁺ T-cell and macrophage activity, cytokine signaling, and fibrosis. Genetic susceptibility and environmental triggers further contribute to disease risk and variability. Clinical presentations span a broad spectrum, ranging from silent conduction abnormalities to ventricular arrhythmias, heart failure, and sudden cardiac death. Diagnostic evaluation integrates electrocardiography, echocardiography, cardiac MRI (CMR), fluorodeoxyglucose positron emission tomography (FDG-PET), and endomyocardial biopsy, supported by the Heart Rhythm Society (HRS), Japanese Ministry of Health and Welfare (JMHW), and World Association of Sarcoidosis and Other Granulomatous Disorders (WASOG) criteria. Management involves immunosuppression with corticosteroids, steroid-sparing agents, and biologics; arrhythmia control through medications, catheter ablation, and device therapy; and guideline-directed heart-failure care, including mechanical support or transplantation when needed. Outcomes remain heterogeneous and depend on ventricular function, fibrosis burden, and timely treatment. Early recognition and coordinated multidisciplinary care are critical to improving survival.

## Introduction and background

Cardiac sarcoidosis (CS) is an inflammatory disease characterized by granulomatous involvement of the myocardium and is associated with increased morbidity and mortality [1,2]. The prevalence of CS is estimated at 10-40 per 100,000 people in the United States and Europe, with African-Americans affected 10-17 times more frequently than Caucasians [2]. Disease pathogenesis is immune-mediated and driven by aberrant CD4⁺ T-cell activation, regulatory T-cell dysfunction, and activated myocardial macrophages, which promote persistent inflammation, granuloma formation, and extracellular matrix degradation through proteolytic enzyme activity [3,4]. Genetic susceptibility, particularly involving HLA class II alleles such as HLA-DQB1*0601, influences the risk of cardiac involvement, clinical severity, and disease phenotype [5]. Early granulomatous infiltration is often clinically silent and may initially manifest as conduction abnormalities, myocardial dysfunction, or ventricular arrhythmias [1].

CS is characterized by progressive myocardial fibrosis, structural remodeling, and electrical instability, which together drive arrhythmia development and ventricular dysfunction [1,6,7]. Involvement of the conduction system commonly results in conduction abnormalities and ventricular arrhythmias, while ongoing myocardial injury leads to heart failure and other cardiac complications [8-12]. Isolated CS may have outcomes similar to systemic disease [13]. However, this remains controversial due to variable findings across studies.

Diagnosis of CS is challenging because presentations are heterogeneous, requiring clinical screening tests followed by advanced imaging (CMR with LGE and T2 sequences, FDG-PET, and CT for extracardiac and coronary assessment), and although biopsy is limited by sampling error, electroanatomic or imaging-guided sampling improves diagnostic yield [8,11-12,14-23]. CS must be distinguished histologically from giant cell myocarditis due to overlapping features [24]. Accordingly, current guidelines for CS endorse a multimodal diagnostic approach to improve diagnostic accuracy [8,25-26].

Management of CS requires a comprehensive strategy addressing inflammation (corticosteroids with steroid-sparing agents such as azathioprine and mycophenolate mofetil, evidence from large randomized controlled trials for these agents is limited, and biologic agents for refractory disease), arrhythmia control (antiarrhythmic drugs, catheter ablation, and device therapy including pacemakers and ICDs), guideline-directed heart-failure treatment, and advanced interventions in selected patients [12,27-33]. Despite therapeutic advances, prognosis remains heterogeneous and is influenced by ventricular function, conduction system disease, myocardial fibrosis burden, and delays in recognition in isolated cases [34-36].

This narrative review summarizes current knowledge on the immunopathogenesis, diagnosis, treatment, and prognosis of CS, highlighting isolated cardiac involvement, risk stratification, and long-term outcomes, addressing the lack of a single review that integrates all these aspects into one comprehensive synthesis.

## Review

Pathophysiology and clinical manifestations of CS

**Figure 1:**
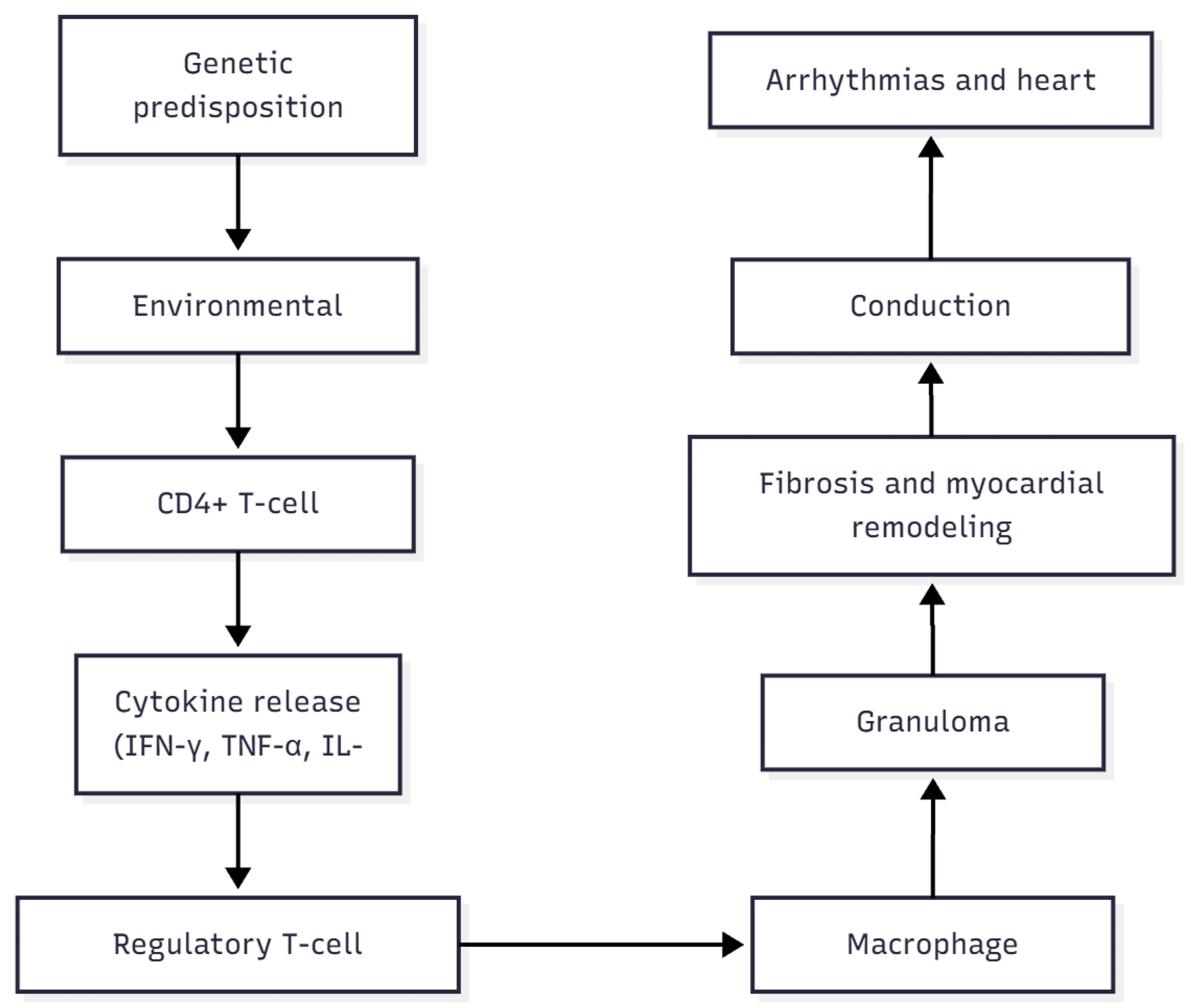
Pathophysiologic Pathway of Cardiac Sarcoidosis

Figure 1 presents a conceptual schematic of the predicted central immunopathogenic pathway involved in CS.

Granulomatous Infiltration and Immune Mechanisms

CS is defined by granulomatous inflammation within the myocardium, causing structural disruption and electrical instability. Histologically, noncaseating granulomas comprise epithelioid cells, multinucleated giant cells, and surrounding lymphocytes. Granulomas are patchily distributed, commonly in the basal interventricular septum, left ventricular free wall, and conduction system, explaining the high prevalence of conduction disturbances [1,2].

Immune dysregulation drives CS pathogenesis. CD4⁺ T-helper cells accumulate at myocardial injury sites and secrete cytokines, such as interferon-gamma, tumor necrosis factor-alpha, and interleukin-2. These recruit and activate macrophages, which differentiate into epithelioid cells forming granulomas [3]. Regulatory T-cell dysfunction permits persistent inflammation and granuloma formation, while activated myocardial macrophages secrete proteolytic enzymes, including matrix metalloproteinases, degrading the extracellular matrix and promoting remodeling [4].

Genetic predisposition plays a key role in determining cardiac involvement in sarcoidosis. Studies have demonstrated that certain HLA class II alleles significantly influence susceptibility, with HLA-DQB1*0601 identified as being associated with an increased risk of CS. This association underscores the importance of antigen presentation pathways in influencing organ-specific myocardial involvement in sarcoidosis. Collectively, these findings support the concept that individual genetic background, particularly genes involved in immune recognition, can influence whether CS develops [5].

Early granulomatous infiltration can be clinically silent. This clinically silent phase may contribute to delayed diagnosis, and the prognosis in asymptomatic CS remains controversial, with some patients remaining stable while others progress to significant dysfunction.

Fibrosis, Remodeling, and Arrhythmogenesis

Chronic granulomatous inflammation in CS frequently progresses to myocardial fibrosis. Activated fibroblasts deposit excessive extracellular matrix proteins, causing ventricular wall stiffening and architectural remodeling [6]. Fibrotic tissue disrupts conduction pathways, creating heterogeneous electrical substrates that predispose patients to reentrant ventricular arrhythmias. The risk of sudden cardiac death is elevated when fibrosis involves the interventricular septum or conduction system [1].

Fibrosis also compromises ventricular contractility, leading to systolic dysfunction, diastolic impairment, and eventual heart failure. Progressive scarring correlates with both the extent of left ventricular dysfunction and the incidence of arrhythmic events. The severity and distribution of fibrosis strongly influence clinical outcomes. Extensive septal or conduction system involvement predicts higher arrhythmic risk, while widespread ventricular fibrosis predisposes to progressive heart failure [7].

Conduction System and Heart Failure Manifestations

CS commonly affects the conduction system, resulting in a spectrum of abnormalities. Involvement of the atrioventricular (AV) node or His-Purkinje system can produce first-degree, second-degree, or complete heart block. Right bundle branch block and other intraventricular conduction delays are frequently observed on electrocardiography and may precede overt cardiac symptoms [8]. Conduction disturbances can be intermittent or progressive, highlighting the need for continuous monitoring in at-risk patients [9].

Ventricular arrhythmias are a hallmark of CS and often arise from areas of granulomatous infiltration or post-inflammatory fibrosis, which create heterogeneous conduction pathways and regions of slow conduction that predispose to reentrant circuits. Both sustained and non-sustained ventricular tachycardia (VT) are frequently observed, and ventricular fibrillation can occur in severe cases [10]. Clinical manifestations range from palpitations and presyncope to syncope and sudden hemodynamic collapse. Electrocardiography may reveal premature ventricular complexes, runs of VT, or conduction abnormalities that correlate with arrhythmic regions [8].

Granulomatous infiltration and progressive fibrosis also impair ventricular contractility, leading to both systolic and diastolic dysfunction. Patients may present with exertional dyspnea, orthopnea, paroxysmal nocturnal dyspnea, and peripheral edema. Heart failure progression correlates with the extent of myocardial involvement and the presence of arrhythmias [11].

Less common manifestations include pericardial effusion, valvular abnormalities, and ventricular aneurysms, which can further compromise cardiac function through secondary valvular incompetence due to papillary muscle dysfunction, ventricular dysfunction, and associated ventricular arrhythmias [2,12].

Isolated vs Systemic Disease

CS may present either as isolated CS (iCS), where inflammation is confined to the heart, or as systemic disease (sCS) with extracardiac involvement. In the 2021 cohort of patients diagnosed using clinical or histological criteria, individuals with iCS showed diagnostic‐criteria features (e.g. AV block, ventricular arrhythmia, septal thinning, left ventricular dysfunction, abnormal myocardial uptake on imaging) that were comparable or more frequent than in sCS [13].

Over a median follow-up of 31 months, 67% of iCS patients experienced the combined outcome of cardiac death, heart‑failure hospitalization, or fatal ventricular arrhythmia, compared with 38% of sCS patients, reflecting a substantially higher event rate in the isolated form. These findings suggest that sarcoidosis confined to the heart can follow an aggressive course comparable to or worse than systemic disease with cardiac involvement, underscoring the clinical relevance of iCS even in the absence of extracardiac signs. However, this study is subject to selection bias, as isolated CS was identified through cardiac symptoms or events, whereas systemic CS may be recognized extracardially and then undergo subsequent cardiac screening, which may result in a higher proportion of less symptomatic or asymptomatic cardiac involvement and more favorable outcomes in the systemic group [13].

Nevertheless, it should be noted that the comparison of outcomes between iCS and sCS remains somewhat controversial, as published studies show heterogeneity in event rates.

Diagnostic approaches in CS

Clinical Evaluation and Initial Screening

Diagnosis of CS is challenging due to its heterogeneous presentation. Early recognition is critical, as timely initiation of therapy improves outcomes. Initial evaluation begins with a detailed clinical history and physical examination, focusing on symptoms such as palpitations, presyncope, exertional dyspnea, and unexplained fatigue. Unexplained syncope or sudden cardiac arrest in a patient with known or suspected sarcoidosis should prompt immediate evaluation for cardiac involvement. Electrocardiography (ECG) is the first-line test. Conduction abnormalities, including AV block, bundle branch block, or ventricular arrhythmias, may be detected even in asymptomatic patients. However, the sensitivity of ECG is low, and normal findings do not exclude disease. Ambulatory monitoring, such as 24-hour Holter or event recorders, increases detection of intermittent arrhythmias and conduction disturbances [8].

Echocardiography is a useful screening tool for detecting structural and functional cardiac abnormalities in patients with suspected CS. Typical findings include regional wall motion abnormalities in non‑coronary distributions, thinning and akinesis of the basal interventricular septum, and aneurysm formation, which may involve the left or right ventricle. Nonetheless, echocardiography lacks sensitivity for detecting early disease and may underestimate myocardial inflammation [14].

Routine laboratory investigations are nonspecific but help exclude alternative etiologies. Serum biomarkers such as angiotensin‑converting enzyme (ACE) and soluble interleukin‑2 receptor (sIL‑2R) may be elevated in sarcoidosis, but their diagnostic utility is limited due to suboptimal sensitivity and specificity [15].

Given these limitations, initial screening tests primarily identify patients who require advanced imaging or biopsy confirmation. A stepwise approach combining clinical suspicion, ECG, echocardiography, and ambulatory monitoring provides a framework for selecting individuals who benefit most from further diagnostic evaluation.

Advanced Imaging Modalities (CMR, PET, CT)

Advanced imaging has become central to the diagnosis of CS, offering noninvasive assessment of myocardial inflammation and fibrosis. Cardiac magnetic resonance imaging (CMR) is a cornerstone modality. Late gadolinium enhancement (LGE) identifies areas of scarring or fibrosis, typically in a patchy, non-coronary distribution involving the basal septum or left ventricular free wall [16]. The presence and extent of LGE strongly correlate with arrhythmic risk and adverse outcomes, making CMR both diagnostic and prognostic [17]. Notably, despite its clinical utility, access to LGE-CMR remains limited in some centers. T2-weighted imaging further detects myocardial edema, reflecting active inflammation [18]. However, midwall enhancement patterns on CMR are not unique to CS and can also appear in other non-ischemic cardiomyopathies, such as arrhythmogenic right ventricular cardiomyopathy [16].

Fluorodeoxyglucose positron emission tomography (FDG-PET) detects metabolically active inflammation in the myocardium. Dietary preparation, such as a high-fat, low-carbohydrate diet, suppresses normal myocardial glucose uptake, thereby enhancing the specificity of FDG-PET for inflammatory lesions [16]. FDG-PET not only aids in diagnosis but also monitors treatment response to immunosuppressive therapy, as reductions in FDG uptake correspond with improved clinical outcomes [19]. Combined CMR and PET imaging increases diagnostic accuracy, allowing simultaneous characterization of inflammation and fibrosis [20].

Computed tomography (CT) is less sensitive for myocardial involvement but plays a role in evaluating extracardiac sarcoidosis, particularly pulmonary or lymph node disease (Table 1). CT coronary angiography may also be useful in excluding ischemic cardiomyopathy when wall motion abnormalities or reduced ejection fraction are present [21].

**Table 1:** Comparative Overview of Advanced Imaging Modalities in Cardiac Sarcoidosis This information was derived from Refs [16,17,21] CMR: Cardiac magnetic resonance imaging; FDG-PET: Fluorodeoxyglucose positron emission tomography

Modality	Primary Purpose	Key Strengths	Limitations
CMR	Assess myocardial fibrosis and inflammation	LGE detects patchy, non-coronary fibrosis in basal septum or LV free wall [16]; T2-weighted imaging identifies myocardial edema [18]; extent of LGE correlates with arrhythmic risk and adverse outcomes [17]	Midwall enhancement is not specific to cardiac sarcoidosis and may appear in other non-ischemic cardiomyopathies, such as arrhythmogenic right ventricular cardiomyopathy [16]
FDG-PET	Detect metabolically active myocardial inflammation	Detects active inflammatory lesions; monitors response to immunosuppressive therapy (reduction in uptake = improvement) [19]; dietary preparation improves specificity [16]	Requires careful dietary preparation to suppress normal myocardial glucose uptake [16]
CT	Evaluate extracardiac sarcoidosis and coronary anatomy	Assesses pulmonary or lymph node involvement; CT coronary angiography helps exclude ischemic cardiomyopathy [21]	Less sensitive for myocardial involvement [21]

Endomyocardial Biopsy (EMB) and Histopathology

EMB remains the histopathological gold standard for diagnosing CS, as demonstration of noncaseating granulomas within myocardial tissue confirms the disease [11]. However, because myocardial infiltration in CS is often focal and patchy, EMB has a low sensitivity of approximately 20 %, and a negative result does not reliably exclude the disease [12].

Sampling error is a major concern, as granulomas often localize to the basal septum or left ventricular free wall, regions not routinely accessible during biopsy [6]. Targeted biopsy techniques guided by electroanatomic voltage mapping and, in some cases, advanced imaging can improve diagnostic yield. Electroanatomic voltage mapping identifies low-voltage myocardium corresponding to scar or inflammation, allowing more precise sampling [22,23].

Histological evaluation requires careful differentiation from giant cell myocarditis, which can mimic sarcoidosis histologically. Giant cell myocarditis typically demonstrates widespread myocardial necrosis and a rapidly progressive clinical course, with multinucleated giant cells present in the absence of granulomas resembling those seen in sarcoidosis [24].

Diagnostic Criteria and Multimodality Integration

Given the limitations of individual diagnostic tools, several professional societies have established criteria that integrate clinical, imaging, and histological findings to improve diagnostic accuracy. The Japanese Ministry of Health and Welfare (JMHW) first proposed guidelines in 1993, later revised in 2017 to incorporate advanced imaging such as FDG-PET and CMR as major criteria [25].

The Heart Rhythm Society (HRS) published consensus criteria in 2014, emphasizing arrhythmias, conduction abnormalities, and imaging findings as key diagnostic components. According to HRS guidelines, a diagnosis can be made with histological confirmation from any organ plus compatible cardiac findings, or with isolated cardiac involvement based on imaging and clinical features [8].

The World Association of Sarcoidosis and Other Granulomatous Disorders (WASOG) has also contributed a diagnostic framework, stratifying organ involvement into “highly probable,” “probable,” and “possible” categories (Table 2) [26].

**Table 2:** Summary Comparison of Diagnostic Criteria Across Major Cardiac Sarcoidosis Guidelines

Society/Organization	Year(s)	Key Diagnostic Focus	Diagnostic Criteria/Notes
Japanese Ministry of Health and Welfare (JMHW)	1993, revised 2017	Integration of clinical, imaging, and histological findings	Guidelines revised to include advanced imaging (FDG-PET, CMR) as major criteria
Heart Rhythm Society (HRS)	2014	Arrhythmias, conduction abnormalities, imaging findings	Diagnosis: histological confirmation from any organ + compatible cardiac findings, or isolated cardiac involvement based on imaging and clinical features
World Association of Sarcoidosis and Other Granulomatous Disorders (WASOG)	Not specified	Organ involvement stratification	Categories: “highly probable,” “probable,” “possible”

Management strategies and future directions

Immunosuppressive Therapy, Corticosteroids, and Steroid-Sparing Agents

Corticosteroids serve as the cornerstone of therapy for CS, particularly in cases exhibiting active myocardial inflammation. High-dose prednisone, typically initiated at 0.5-1 mg/kg/day based on expert consensus, is employed to effectively control this inflammation. Following the acute phase, a tapered maintenance regimen is implemented, guided by clinical symptoms and imaging findings. The duration and specific dosing of corticosteroid therapy are tailored to the individual patient's response and tolerance. While corticosteroids are the primary treatment modality, their use is often complemented with steroid-sparing agents to mitigate potential side effects and enhance long-term management outcomes. This comprehensive approach underscores the importance of a personalized treatment plan to optimize the management of CS [27].

Long-term corticosteroid therapy in CS is associated with potential adverse effects, including an increased risk of infection, which has prompted consideration of steroid-sparing immunosuppressive agents. Among these, methotrexate is the most commonly used and has observational evidence suggesting it may allow reduction of corticosteroid doses and help maintain disease control. Azathioprine and mycophenolate mofetil are also described as alternative immunosuppressive options for patients in whom methotrexate is not suitable [12,28]. However, large prospective randomized controlled trials evaluating these agents in CS are lacking, which is an important limitation to note.

Biologic agents, particularly the TNF-α inhibitor infliximab, are considered for patients with CS who are refractory to conventional therapy. Infliximab has demonstrated efficacy in selected treatment-resistant patients, controlling disease activity and enabling corticosteroid dose reduction [29]. Although TNF-α inhibitors carry a theoretical risk of worsening heart failure, a retrospective, single-center study of 77 patients found that 20 treated with TNF‑α inhibitors showed improvement or resolution of imaging abnormalities, stable or improved ventricular function, and reduced prednisone doses [30].

Arrhythmia Management: Drugs, Ablation, and Device Therapy

Arrhythmia management in CS aims to prevent sudden cardiac death and control symptomatic arrhythmias. Antiarrhythmic drugs, especially class III agents such as amiodarone, are commonly used as adjunctive therapy, though their effectiveness may be reduced in the presence of myocardial scarring. Long-term therapy carries potential organ toxicity, and drug choice should account for comorbidities and careful monitoring. Beta-blockers and guideline-directed heart-failure medications are often used adjunctively to optimize myocardial substrate, while device therapy or ablation is considered in drug-refractory cases [8,11].

Catheter ablation targets recurrent VT when pharmacologic therapy fails. Ablation outcomes are heterogeneous because the arrhythmogenic substrate is often intramural or multifocal. While ablation frequently reduces VT burden, recurrence rates remain common [31].

Implantable cardioverter-defibrillators (ICDs) are recommended for survivors of cardiac arrest, sustained VT, or significantly reduced LVEF despite therapy. Many experts also favor lower thresholds for ICD placement in CS due to the unpredictable arrhythmic risk and progressive scarring. Pacemaker implantation is indicated for symptomatic high-grade AV block, while cardiac resynchronization therapy (CRT) may benefit selected patients with reduced EF and electrical dyssynchrony. Device selection must also account for infection risk in immunosuppressed patients and long-term surveillance for appropriate therapy [8,32].

Heart Failure Management and Advanced Therapies

Heart failure therapy in CS follows guideline-directed medical therapy (GDMT): ACE inhibitors/ARBs/ARNI, beta-blockers, mineralocorticoid receptor antagonists, and SGLT2 inhibitors where indicated [33]. When standard medical and device therapies are insufficient, advanced interventions such as mechanical circulatory support or heart transplantation should be considered. Ventricular assist devices may serve as a bridge to transplant in selected patients; however, active systemic sarcoidosis and heightened infection risk complicate candidacy and require careful multidisciplinary evaluation. Heart transplantation is an option for refractory, end-stage CS; outcomes are acceptable when extracardiac disease is controlled and immunologic risk is managed. Pre-transplant assessment must evaluate extracardiac sarcoidosis activity, infection screening, and immunologic compatibility [12].

Prognosis and outcomes

Recent studies provide a more hopeful outlook for CS than older data, but risks remain, especially for certain subgroups. In a large multicenter observational study of 157 patients followed over a median of seven years, overall survival at five years was ~93.6% (95% CI: 89.5-97.8), and at 10 years ~89.6% (95% CI: 83.8-95.8). Key baseline predictors of higher mortality included reduced left ventricular ejection fraction (LVEF <40%), presence of high-degree AV block at presentation, older age, hypertension, abnormal pulmonary function tests, and LGE on cardiac MRI [34].

Event-free survival, defined as survival without death, transplant, or durable mechanical circulatory support, shows a steeper decline over time. In a recent cohort with a median follow-up of 9.7 years, event-free survival rates were 96% at one year, 79% at five years, and 58% at 10 years. Outcomes did not differ significantly between patients with isolated CS (iCS) and those with systemic sarcoidosis involving the heart when measured against this composite endpoint [35].

Relapse is another major concern. In the PLOS ONE study, 10-year relapse-free survival for cardiac relapses alone was approximately 53% (95% CI: 44-63). When both cardiac and non-cardiac relapses were combined, relapse-free survival dropped to ~27.4% at 10 years. Independent predictors of higher cardiac relapse risk included impaired kidney function, echocardiographic wall motion abnormalities, and left heart failure, whereas cutaneous involvement correlated with lower relapse risk [34].

Reduced LVEF (<40%), older age, and high-degree AV block at diagnosis are independent predictors of worse long-term survival in patients with CS. Baseline functional status and quality-of-life measures were recorded, but longitudinal data on progression of New York Heart Association (NYHA) functional class or patient-reported outcomes were limited [34].

Regional differences and diagnostic delays also influence prognosis. Patients with iCS often present later and with more arrhythmic events, likely due to delayed recognition. Despite these differences at diagnosis, long-term survival and event-free survival appear broadly similar between iCS and systemic CS cohorts once diagnosis is established [35]. As mentioned earlier, the comparison of outcomes between iCS and sCS remains debated, as studies report heterogeneous event rates.

Stratification by advanced heart failure (AHF) status further refines prognostic assessment. In a UK study population, CS patients with LVEF <50% who met ESC-AHF criteria (which define AHF as persistent severe symptoms despite optimal medical therapy [36]) had significantly higher rates of adverse composite outcomes (including mortality, urgent transplant, or durable device support) compared with patients not meeting AHF criteria [37].

In summary, prognosis in CS is heterogeneous. While many patients survive more than a decade after diagnosis (particularly younger patients with preserved LVEF and limited myocardial scarring), a sizeable proportion experiences relapse or progression. Patients with reduced systolic function, conduction abnormalities, or AHF features remain at the highest risk.

## Conclusions

CS remains a clinically challenging condition with variable presentations and outcomes. Despite advances in diagnostic imaging, histopathology, and immunomodulatory therapies, early detection and risk stratification are essential to prevent adverse events, including ventricular arrhythmias, heart failure, and sudden cardiac death. Tailored management integrating immunosuppression, arrhythmia control, and heart failure interventions improves long-term survival, particularly in patients identified before significant myocardial remodeling occurs. Future research focusing on standardized diagnostic criteria, biomarkers, and targeted therapies promises to refine treatment strategies, reduce relapse rates, and enhance quality of life. Multidisciplinary care and individualized therapy remain central to optimizing outcomes in this complex disease.
